# Primary mitral valve regurgitation: Update and review

**DOI:** 10.21542/gcsp.2017.3

**Published:** 2017-03-31

**Authors:** Eirini Apostolidou, Andrew D Maslow, Athena Poppas

**Affiliations:** 1 Brown University, Section of Cardiology; 2 Section of Cardiac Anesthesia, Rhode Island and Miriam Hospital, Providence, RI, USA

## Abstract

Mitral regurgitation is the second most common valvular disorder requiring surgical intervention worldwide. This review summarizes the current understanding of primary, degenerative mitral regurgitation with respect to etiology, comprehensive assessment, natural history and management. The new concept of staging of the valvular disorders, newer predictors of adverse and controversy of “watchful waiting” versus “early surgical intervention” for severe, asymptomatic, primary mitral regurgitation are addressed.

## Introduction

The retrograde flow of blood from the left ventricle through the mitral valve into the left atrium defines mitral regurgitation. Mitral regurgitation is the most common valvular disorder in the United States, affecting more than 2 million individuals, with a striking increase in prevalence with advanced age.^1,2^ From a population-based study, the prevalence of mitral regurgitation is greater than 10% in adults older than 75 years, with no significant difference in age-adjusted rates between men and women.^2^ In the Euro Heart Survey, moderate or severe mitral regurgitation requiring surgical intervention was the second most common form of valvular abnormality, behind only aortic stenosis (mitral regurgitation 31.5% versus aortic stenosis 43.1%).^3^

This review will highlight the etiology, pathophysiology and natural history of primary mitral valve regurgitation. Predictors of adverse outcomes will be described followed by discussion of treatment and timing of surgery, the latter delving into the controversy between “watchful waiting” versus “early surgical intervention” for severe, asymptomatic primary mitral regurgitation.

## Mechanisms and Causes of Mitral Regurgitation

Mitral regurgitation can occur due to disease of the mitral valve leaflets and/or abnormalities of the mitral valve apparatus or secondary to left ventricular dysfunction. Functionally the mitral valve apparatus consists of several components;^4,5^

- The mitral annulus
- The anterior and posterior mitral valve leaflets
- The chordae
- The anterolateral and posteromedial papillary muscles
- The left ventricular myocardium underlying the papillary muscles

Dysfunction or altered anatomy of any of these components can lead to mitral regurgitation. The mechanism of mitral regurgitation may be described as primary or secondary. Primary mitral regurgitation, sometime called degenerative or organic, is due to an intrinsic lesion of the mitral valve apparatus. Secondary mitral regurgitation, sometimes called functional or ischemic, is a disease of the left ventricle; the left ventricular remodeling in dilated cardiomyopathy or the segmental wall motion abnormalities in ischemic cardiomyopathy, can displace the papillary muscles apically and laterally, causing tethering and malcoaptation of the mitral valve leaflets, which leads to secondary mitral regurgitation.^6^

**Figure 1.**
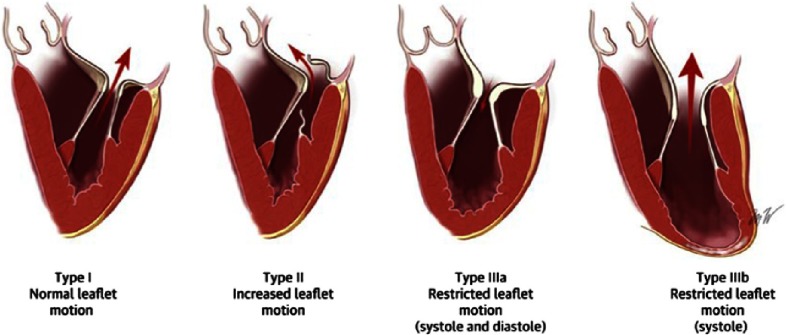
Mitral valve anatomy and Carpentier Classification of mitral regurgitation (from ref # [32]).

**Figure 2.**
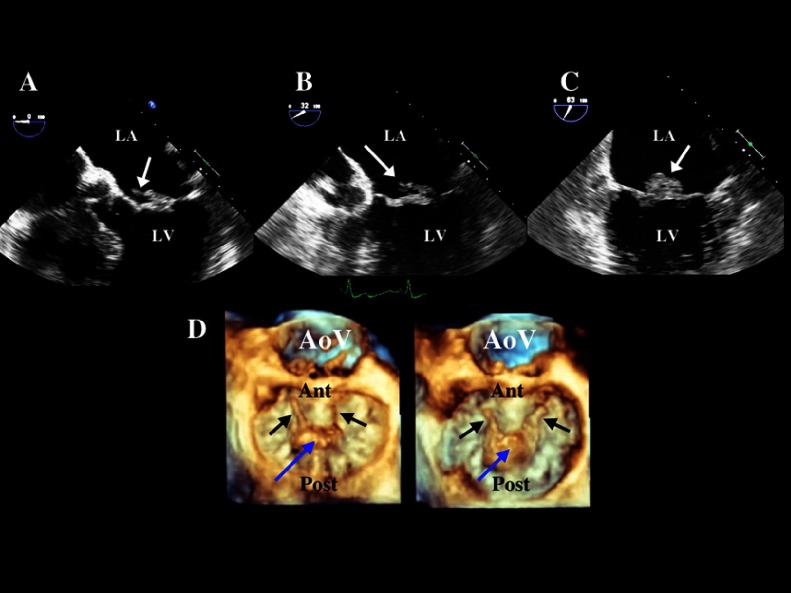
The TEE images demonstrates fibroelastic deficiency with prolapse and flail of P2 Scallop with torn chordae (white arrows) in 2D imaging in three different mid-esophageal TEE views (panel A, B, C) and in 3D imaging (blue arrow = P2 scallop prolapse) (black arrows = torn chordae).
- There are two major phenotypes of the degenerative mitral valve disease/prolapse: a) fibroelastic deficiency and b) Barlow’s disease.^13,14^ Fibroelastic deficiency is usually seen in individuals older than 60 years. It is often characterized by single chordal rupture and prolapse of an isolated scallop, most commonly the P2. The associated mitral regurgitation jet is usually eccentric and directed opposite to the prolapsing scallop^17^ (Movie 1) Barlow’s disease is typically seen in younger patients, 40–60 years old, who present with a chronic murmur. It is characterized by excess leaflet tissue throughout. The leaflets and the chordae appear thickened, redundant and elongated. Multiple scallops of both anterior and posterior leaflets prolapse or may flail into the left atrium during systole^15,16^ (Movie 2). Of note, these 2 forms of mitral valve prolapse represent the two ends of a spectrum. In clinical practice, most of the patients fall between these two extremes.

For surgical intervention, the Carpentier Classification is used to group the causes of mitral regurgitation into 3 types, based on the mobility of the mitral valve leaflets [Figure 1]^7^:

- **Carpentier type I mitral regurgitation** is characterized by normal leaflet mobility. The mitral regurgitation is usually due to a dilated mitral annulus and less often due to a perforated leaflet from endocarditis.^8^ In normal adults, the mitral annulus is soft and flexible and its contraction contributes significantly to the mitral valve closure. Mitral regurgitation due to annular dilation can occur in any form of heart disease associated with dilation of the left ventricle and especially dilated cardiomyopathy. The annular dilation occurs primarily along the posterior annular section and the associated mitral regurgitation jet is most often centrally directed.^8^
- **Carpentier type II mitral regurgitation** is characterized by increased leaflet mobility and it is usually due to leaflet or chordae pathology. The most common cause of primary mitral regurgitation is degenerative mitral valve disease,^9,10^ predominately mitral leaflet prolapse and/or flail.^11,12^ (Figure 2)

**Movie 1.**
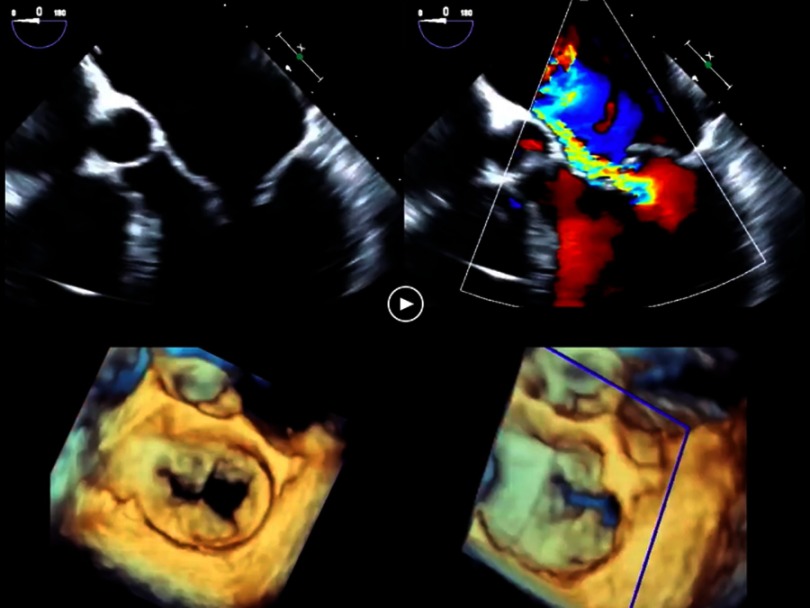
The TEE demonstrates fibroelastic deficiency with prolapse and flail of the P2 scallop shown in 2D imaging (upper left), with color Doppler of the eccentric mitral regurgitation (upper right) and 3D TEE imaging en face from left atrium (lower right) and tilted laterally (lower left). (“Movie files are available at https://globalcardiologyscienceandpractice.com ”)

**Movie 2.**
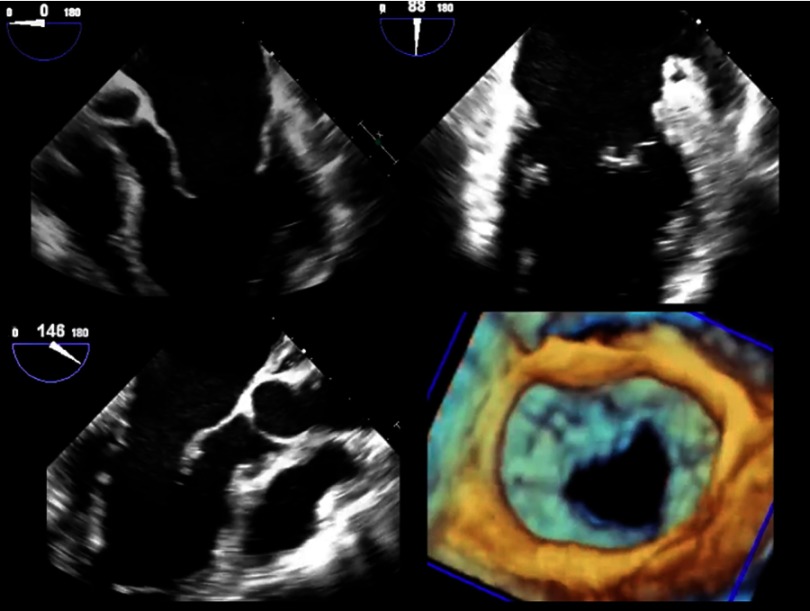
The TEE demonstrates bi-leaflet mitral valve prolapse (Barlow’s disease) shown in 2D imaging in three different mid-esophageal views (upper right, upper left, lower right panels) and 3D en face view from the left atrium (lower left panel). (“Movie files are available at https://globalcardiologyscienceandpractice.com ”)
- **Carpentier type III mitral regurgitation** is the result of reduced leaflet mobility and it is further classified to types IIIa and IIIb. In type IIIa mitral regurgitation the leaflet mobility is reduced in both systole and diastole. This is usually seen in rheumatic valve disease or as a result of radiation therapy. Rheumatic mitral regurgitation is characterized by some degree of commissural fusion, but chordal fusion and shortening and leaflet retraction are more prominent findings.^18^ Similar pathology is noted in post- radiation mitral regurgitation. ^19^ In type IIIb, the mitral regurgitation is due to myocardial ischemia and/or ventricular remodeling, while the leaflets appear anatomically normal, with reduced mobility during systole.^20,21^ The cause is papillary muscle displacement with apical tethering and loss of coaptation of the leaflets.

## Pathophysiology of Chronic Mitral Regurgitation

Mitral regurgitation results in left ventricular volume overload, due to an increase in the total stroke volume, as blood is ejected both forward into the aorta and backward into the left atrium. The compensatory response is hypertrophy of the myocardium, progressive dilation and increase in the left ventricular end- diastolic volume, with initial normalization of the wall stress. Long- standing mitral regurgitation causes progressive left ventricular dilation and decline in the left ventricular contractility and ejection fraction. These structural and functional changes, may be clinically silent and precede functional limitations and symptoms. The left atrium also gradually dilates and its compliance increases, in an attempt to maintain normal left atrial pressure. Eventually an increase in the left atrial and left ventricular diastolic pressures and an increase in pulmonary vascular resistance cause the symptoms of heart failure and clinical decompensation.^22–24^

## Presentation

Most patients with severe, chronic mitral regurgitation remain asymptomatic for many years, due to the compensatory pathophysiologic mechanisms described above. Symptoms of dyspnea and heart failure eventually develop as the compensatory mechanisms begin to fail and left ventricular dilation and systolic dysfunction occur.^25^ Symptoms may occur in patients with preserved left ventricular function who have elevated pulmonary venous pressures or develop atrial fibrillation. By the time the symptoms due to reduced cardiac output and/ or pulmonary congestion become apparent, serious and sometimes irreversible left ventricular dysfunction has occurred.^26^

## Diagnosis and evaluation of mitral regurgitation

Echocardiography plays a pivotal role in the diagnosis of mitral valve regurgitation, the determination mechanism/ cause, the quantification of its severity, and its effect/ consequences on the left ventricle.^27^ Once defined, further imaging data is used to determine prognosis, timing of surgical intervention and feasibility of successful surgical repair.

The underlying cause of the mitral regurgitation, such as mitral valve prolapse, chordal rupture, can be often determined by transthoracic echocardiography but detailed analysis requires transesophageal.^5,28^ Transthoracic echocardiography can differentiate primary from secondary regurgitation and provide anatomic information that support repair over replacement of the valve.^29,30^

**Figure 3.**
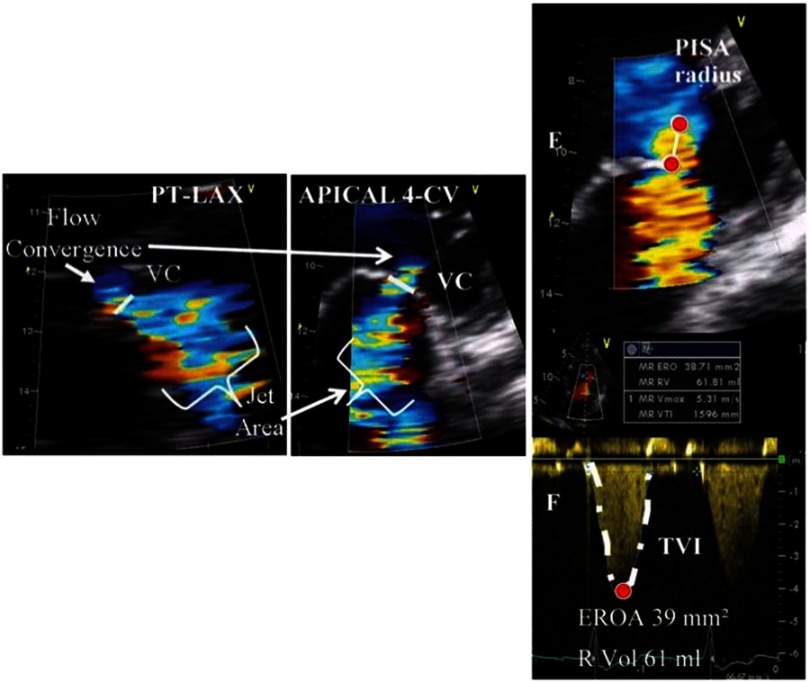
(A) (left panel): Assessment of mitral regurgitation using the vena contracta width (from ref [32]). (B) (right panel): Quantitative assessment of mitral regurgitation using the Proximal Isovelosity Surface Area method (PISA) (from [32]).

Doppler echocardiography provides significant information on the severity of the mitral regurgitation. Qualitative assessment of the regurgitant jet area using color flow Doppler is influenced by the cause of the regurgitation and the jet eccentricity and it should not be used alone for the grading of the lesion severity.^31^ Quantitative methods, which measure the regurgitant volume, the regurgitant fraction and the effective regurgitant orifice area (EROA) appear to have greater accuracy and are currently recommended by the ASE guidelines and the European Association of Echocardiography.^27,32^ Quantification of the EROA can be performed by using the Proximal Isovelocity Surface Area (PISA) (Figure 3A)^33,34^ or by calculation of the aortic and mitral stroke volumes.^35^ Severe primary mitral regurgitation is diagnosed with an EROA of 40 mm^2^, while a smaller EROA ≥20 mm^2^ is consistent with severe mitral regurgitation in patients with ischemic disease.^27^ The vena contracta, defined as the narrowest portion of the regurgitant jet, also predicts the severity of mitral regurgitation (Figure 3B).^36^ Flow reversal in the pulmonary veins and high peak mitral inflow velocity support the diagnosis of severe mitral regurgitation (Table 1).^37^

**Table 1:** Qualitative and quantitative parameters useful in grading mitral regurgitation severity (from [27]).

	Mild	Moderate		Severe
**Structural parameters**				
LA size	Normal^*^	Normal or dilated		Usually dilated^**^
LV size	Normal^*^	Normal or dilated		Usually dilated^**^
Mitral leaflets or support apparatus	Normal or abnormal	Normal or abnormal		Abnormal/ Flail leaflet/ Ruptured papillary muscle
**Doppler parameters**				
Color flow jet area^*ζ*^	Small, central jet (usually <4 cm^2^ or <20% of LA area)	Variable		Large central jet (usually >10 cm^2^ or >40% of LA area) or variable size wall- impinging jet swirling in LA
Mitral inflow–PW	A wave dominant^ϕ^	Variable		E wave dominant^ϕ^ (E usually 1.2 m/s)
Jet density–CW	Incomplete or faint	Dense		Dense
Jet contour –CW	Parabolic	Usually parabolic		Early peaking–triangular
Pulmonary vein flow	Systolic dominance^§^	Systolic blunting^§^		Systolic flow reversal^†^
**Quantitative parameters^ψ^**				
VC width (cm)	<0.3	0.3–0.69		≥0.7
R Vol (ml/beat)	<30	30–44	45–59	≥60
RF (%)	<30	30–39	40–49	≥50
EROA (cm^2^)	<0.20	0.20–0.29	0.30–0.39	≥0.40

**Notes.**

CW Continuous wave LA left atrium EROA effective regurgitant orifice area LV left ventricle PW pulsed wave RF regurgitant fraction R Vol regurgitant volume VC vena contracta

* Unless there are other reasons for LA or LV dilation. Normal 2D measurements: LV minor axis ≤2.8 cm/m^2^, LV end-diastolic volume ≤82 ml/m^2^, maximal LA antero-posterior diameter ≤2 cm/m^2^, maximal LA volume ≤36 ml/m^2^ (2,33,35).

** Exception: acute mitral regurgitation.

ζ At a Nyquist limit of 50–60 cm/s.

† Pulmonary venous systolic flow reversal is specific but not sensitive for severe MR.

ϕ Usually above 50 years of age or in conditions of impaired relaxation, in the absence of mitral stenosis or other causes of elevated LA pressure.

§ Unless other reasons for systolic blunting (eg. atrial fibrillation, elevated left atrial pressure).

ψ Quantitative parameters can help sub-classify the moderate regurgitation group into mild-to-moderate and moderate-to-severe.

Transthoracic echocardiography is also very helpful in the follow-up of patients with mitral regurgitation. Based on the stage of the disease, an echocardiogram is repeated every 3–5 years for Stage A, every 1–2 years for Stage B, and every 6–12 months for stage C mitral regurgitation.^6^

Exercise echocardiography has its role in the evaluation of mitral regurgitation, by providing information on the severity of the regurgitation and the hemodynamic abnormalities (e.g., pulmonary hypertension) during exercise.^38^ It is a useful tool to evaluate symptoms in patients that appear to have only mild regurgitation, to determine the functional capacity^39^ and the changes in hemodynamics in patients who appear stable or asymptomatic.^40^

Cardiac MRI is the most accurate non- invasive technique for measurement of end- diastolic and end-systolic volumes and left ventricular mass. Although visualization of mitral valve structure is more reliable by echocardiography, CMR may provide a more accurate assessment of the severity of regurgitation.^41^ In patients in whom discrepancy exists between mitral regurgitation severity by clinical findings and echo results, further evaluation with CMR may be helpful.^42^

Left heart catheterization, with coronary angiography and ventriculography is indicated in the following circumstances in the evaluation of mitral regurgitation: a) discrepancy between clinical findings and echocardiographic data, b) detection and severity assessment of associated valvular lesions and c) presence and extent of coronary artery disease, especially in preparation for surgical intervention.^6^

## Stages of Mitral Regurgitation

The 2014 ACC/ AHA valve guidelines introduced stages for each valve lesion,^6^ which are similar to the stages proposed in the Heart Failure guidelines. Each stage describes the progression of the valvular disorder taking into account the presence or absence of symptoms, the severity of the valve disorder, the response of the left ventricle to the effect of the disorder, the effect on pulmonary circulation and heart rhythm. The staging system provides the physician a better way to monitor the progression of the valvular disease, defines the clinical and echocardiographic follow-up and helps make management decisions based on the stage of the disorder in order to treat complications and effects of the disorder in a more timely fashion. Table 2 provides a summary of the stages proposed for primary mitral regurgitation.

**Table 2:** Stages of primary mitral regurgitation (reproduced from [6]).

Grade	Definition	Valve Anatomy	Valve Hemodynamics^*^	Hemodynamic Consequences	Symptoms
A	At risk of MR	• Mild mitral valve prolapse with normal coaptation • Mild valve thickening and leaflet restriction	• No MR jet or small central jet area <20% LA on Doppler • Small vena contracta <0.3 cm	• None	• None
B	Progressive MR	• Severe mitral valve prolapse with normal coaptation • Rheumatic valve changes with leaflet restriction and loss of central coaptation • Prior IE	• Central jet MR 20%-40% LA or late systolic eccentric jet MR • Vena contracta <0.7 cm • Regurgitant volume <60 mL • Regurgitant fraction <50% • ERO <0.40 cm^2^ • Angiographic grade 1–2+	• Mild LA enlargement • No LV enlargement • Normal pulmonary pressure	• None
C	Asymptomatic severe MR	• Severe mitral valve prolapse with loss of coaptation or flail leaflet • Rheumatic valve changes with leaflet restriction and loss of central coaptation • Prior IE • Thickening of leaflets with radiation heart disease	• Central jet MR >40% LA or holosystolic eccentric jet MR • Vena contracta ≥0.7 cm • Regurgitant volume ≥60 mL • Regurgitant fraction ≥50% • ERO 0.40 cm^2^ • Angiographic grade 3–4+	• Moderate or severe LA enlargement • LV enlargement • Pulmonary hypertension may be present at rest or with exercise • **C1**: LVEF >60% and LVESD <40 mm • **C2**: LVEF ≤60% and LVESD ≥40 mm	• None
D	Symptomatic severe MR	• Severe mitral valve prolapse with loss of coaptation or flail leaflet • Rheumatic valve changes with leaflet restriction and loss of central coaptation • Prior IE • Thickening of leaflets with radiation heart disease	• Central jet MR >40% LA or holosystolic eccentric jet MR • Vena contracta ≥0.7 cm • Regurgitant volume ≥60 mL • Regurgitant fraction ≥50% • ERO 0.40 cm^2^ • Angiographic grade 3–4+	• Moderate or severe LA enlargement • LV enlargement • Pulmonary hypertension present	• Decreased exercise tolerance • Exertional dyspnea

**Notes.**

* Several valve hemodynamic criteria are provided for assessment of MR severity, but not all criteria for each category will be present in each patient. Categorization of MR severity as mild, moderate, or severe depends on data quality and integration of these parameters in conjunction with other clinical evidence.

ERO effective regurgitant orifice IE infective endocarditis LA left atrium/atrial LV left ventricular LVEF left ventricular ejection fraction LVESD left ventricular end-systolic dimension MR mitral regurgitation

## Natural History, Progression and Predictors of Outcomes in Mitral Regurgitation

The natural history of organic mitral regurgitation is highly variable and depends on a combination of parameters that include the regurgitant volume, the cause of the regurgitation and its effect on the left ventricle. Asymptomatic patients with mild primary mitral regurgitation usually remain stable for many years. Severe mitral regurgitation develops only is a small percentage of these patients, due to intervening infective endocarditis or chordal rupture.

Different series examined the natural history of patients with severe mitral regurgitation. Early series reported widely variable mortality rates, ranging from 27% to 97% at 5-year follow-up.^43,44^ That variation may be explained by poorly defined severity of mitral regurgitation, various selection biases and small study populations.^43–48^ A series from Ling *et al.* examined 229 patients with mitral regurgitation due to flail leaflet, many of who were symptomatic, had atrial fibrillation or evidence of left ventricular dysfunction.^49^ Patients who were treated medically had a mortality rate significantly higher than the expected (6.3% yearly mortality, p = 0.016, when compared with the expected rate in the US population according to the 1990 census). Death or need for surgery was almost unavoidable within 10 years of diagnosis and surgical correction improved long- term survival.^49,50^

Two recent series involved patients with mitral regurgitation who were initially asymptomatic and had a normal left ventricular function.^51,52^ Enriquez-Sarano *et al.* examined prospectively 456 patients with asymptomatic organic mitral regurgitation and showed that the 5-year mortality from any cause was 22% and the cardiac mortality was 14% in patients managed medically. Cardiac surgery was ultimately performed in 232 patients and was associated with improved survival.^51^ Rosenhek *et al.* followed a series of 132 asymptomatic patients with severe degenerative mitral regurgitation. Survival without the need of surgery was 92% at 2 years, 78% at 4 years and 65% at 6 years. A total of 38 patients developed indications for surgery and those with a flail leaflet tended to develop criteria for surgery slightly, but not significantly earlier.^52^

Despite the lack of randomized trials, all the prospective, observational data showed that in asymptomatic patients with initially preserved ejection fraction, severe mitral regurgitation has a high likelihood of requiring surgery over the next 6–10 years, because of heart failure symptoms or left ventricular dysfunction.^53,54^

Many predictors of different clinical outcomes and especially mortality have been identified in patients with primary mitral regurgitation. Ling *et al.* showed that in patients with mitral regurgitation due to flail leaflets, older age, presence of symptoms and lower ejection fraction are independent predictors of mortality.^50^

Enriquez-Sarano *et al.* demonstrated that the Effective Regurgitant Orifice Area (EROA) is a powerful predictor of outcomes in patients with asymptomatic, organic mitral regurgitation.^51^ When compared to patients with EROA < 20 mm^2^, those with an orifice of at least 40 mm^2^ have an increased risk of death from any cause, death from cardiac causes and cardiac events (defined as death from cardiac causes, heart failure and new atrial fibrillation).

Tourneau *et al.* examined the impact of left atrial volume on clinical outcomes in 492 patients with organic mitral regurgitation and showed that the left atrial index is a predictor of long-term outcomes.^55^ Patients with a left atrial index ≥60 ml/m^2^ have lower 5-year survival and more cardiac events than those with mild or no left atrial enlargement. In this cohort, mitral surgery is associated with decreased mortality and cardiac events.^55^

The MIDA registry included patients with mitral regurgitation due to flail leaflets.^56^ Pulmonary artery systolic pressure was measured by echocardiography is 437 patients and pulmonary hypertension was observed in 102 patients. Pulmonary hypertension is an independent predictor of all- cause death (adjusted HR: 1.70, p = 0.002), cardiovascular death (adjusted HR: 2.21, p = 0.003) and heart failure (adjusted HR: 1.70, p = 0.002).^56^ In that registry, mitral valve surgery was beneficial, but it didn’t abolish completely the effects of pulmonary hypertension once it was established.

Atrial fibrillation is a common arrhythmia in patients with chronic mitral regurgitation and its onset is a marker of disease progression.^57^ Patients with atrial fibrillation have an adverse outcome compared to those who remain in sinus rhythm^58^ and the development of atrial fibrillation is considered an indication (IIa) for surgical intervention.^6^

Over the past few years new prognostic markers have emerged. Those include the b- natriuretic peptide (BNP), the use of left ventricular strain^59^ and the exercise capacity.^60^

BNP activation in organic mitral regurgitation is primarily due to ventricular and atrial consequences, rather than the degree of mitral regurgitation.^61^ Higher BNP levels are associated with lower survival and higher combined adverse events (death and heart failure).^62^

Alashi *et al.* examined 448 asymptomatic patients with severe primary mitral regurgitation and preserved ejection fraction and demonstrated that abnormal longitudinal strain and higher BNP levels are associated with higher long-term mortality and the combination of two appeared to be a synergistic outcome predictor.^63^

The importance of exercise capacity in predicting outcomes in patients with severe primary regurgitation was studied by Naji *et al.*^64,65^ In 576 patients with primary mitral regurgitation who underwent exercise echocardiography prior to mitral valve surgery, lower achieved METs were associated with worse long-term outcomes. The authors concluded that achieving >100% of age and gender- predicted METs can safely delay mitral valve surgery for at least one year, without an effect on outcomes.^65^

Kusunose *et al.* studied 196 patients with moderate to severe, primary asymptomatic mitral regurgitation and showed that resting left ventricular strain, exercise TAPSE and exercise systolic pulmonary arterial pressure are independent predictors of time to surgery.^66^ Exercise- induced right ventricular dysfunction is an independent predictor of worse outcomes in in this patient cohort. Table 3 summarizes the clinical, biologic and echocardiographic predictors of poor outcome in patients with primary mitral regurgitation.

**Table 3:** Predictors of poor outcome in primary mitral regurgitation.

Clinical Characteristics	Biologic Markers	Echo Findings
Advance age	Elevated BNP	Low ejection fraction (<60%)
Symptoms of CHF		EROA (>40 mm^2^)
Atrial fibrillation		Left atrial volume
Poor exercise capacity		Pulmonary hypertension
		Abnormal LV strain

## Management of Mitral Regurgitation

### A. Medical management of primary mitral regurgitation

No medical therapy alters the natural history of severe primary mitral regurgitation. Medical therapy for systolic dysfunction, which includes beta-blockers, ACE-I and possibly aldosterone antagonists, is reasonable (Class IIa recommendation) in symptomatic patients with primary mitral regurgitation, who have a left ventricular ejection fraction<60% and in whom surgery is not planned or it will be delayed.^6^ Diuretics may relieve the symptoms of heart failure, but improvement in symptoms should not delay referral for surgical intervention. If the left ventricular systolic function is normal vasodilator therapy is not indicated for asymptomatic, normotensive patients with chronic primary regurgitation.^6^ A limited number of studies addressed the use of ACE-I therapy for 1–6 months in chronic asymptomatic mitral regurgitation with preserved systolic function. These studies failed to provide evidence of clinical or hemodynamic benefit.^67–71^ Hypertension needs to be treated, because the increased left ventricular systolic pressure increases the trans-mitral gradients and worsens the severity of mitral regurgitation.^6^

### B. Indications for surgical intervention in primary mitral regurgitation

Surgical intervention with either mitral valve repair or replacement is indicated in patients with severe mitral regurgitation who develop symptoms or left ventricular dysfunction.^6,72^ The left ventricular dysfunction is defined as an ejection fraction <60% and/or an end- systolic dimension >40 mm (Class I recommendations). Concomitant mitral valve repair or replacement is also indicated in patients with chronic severe primary mitral regurgitation undergoing cardiac surgery for another indication (Class I recommendation).^6,72^

Mitral valve repair is reasonable in asymptomatic patients with chronic severe primary mitral regurgitation (Stage C1) and preserved systolic function in whom the likelihood of successful repair is >95% with an expected mortality <1% (Class IIa recommendation).^6^ Another reasonable indication for mitral valve repair is in asymptomatic patients with chronic severe primary mitral regurgitation (Sage C1) with new onset atrial fibrillation or resting pulmonary hypertension (Class IIa recommendation).^6,72^

**Figure 4.**
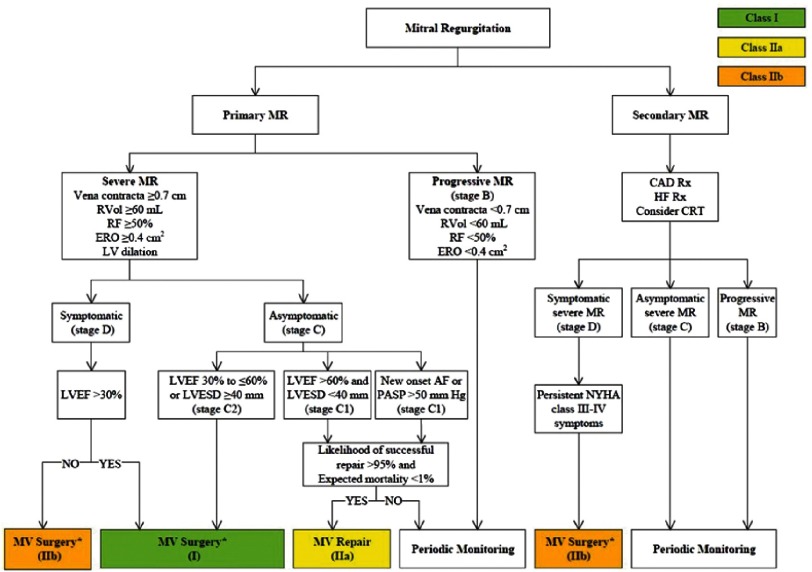
Summary of the indications for surgical intervention in mitral regurgitation as per the 2014 AHA/ACC valve guidelines (from [6]).

A summary of the recommendations for surgical intervention in primary and functional mitral regurgitation as per the 2014 ACC/AHA valve guidelines is shown below (Figure 4). These recommendations are basically similar to the European valve guidelines, with a couple of caveats: When there is a high likelihood of durable repair at a low surgical risk, Vahanian et al. recommend valve repair in patients with a flail leaflet and LVESD ≥40 mm (Class IIa), while surgery may be considered (Class IIb) if one of the following risk factors is present: left atrial volume ≥60 ml/m3 BSA and sinus rhythm or pulmonary hypertension with exercise (SPAP ≥60 mmHg).^72^

### C. Mitral valve repair versus replacement

Mitral valve repair is the preferred treatment for patients with primary mitral regurgitation as it is associated with better outcomes than mitral valve replacement.^73–76^ Surgical repair of the valve tends to be successful in the following cases: degenerative mitral valve disease, annular dilation, chordal rupture, leaflet perforation due to endocarditis and papillary muscle dysfunction due to ischemia. It is less likely to be successful in older patients with calcified deformed valves, rheumatic heart disease or severe sub-valvular thickening. These cases are usually treated with mitral valve replacement.^77–79^

There are certain disadvantages associated with the mitral valve replacement that have made it a less favorable strategy. The left ventricular function and the ejection fraction tend to deteriorate after mitral valve replacement, contributing to early and late morbidity and mortality.^80,81^ That appears to be associated with the loss of support of the mitral valve apparatus, as chordae and papillary muscles are not preserved with valve replacement.^82^

Another disadvantage of the valve replacement is the prosthesis itself. Mechanical prostheses required life-long anticoagulation and they are associated with an increased risk of thrombosis, bleeding and thromboembolism. Bioprostheses are associated with late structural deterioration and need for repeat intervention. Both types of prostheses have an increased risk of infective endocarditis.^6,72^

For all these reasons efforts are being made to repair a mitral valve whenever possible. Mitral valve repair is technically a more demanding procedure than replacement and there is a tendency to refer patients to centers of excellence in performing mitral valve repair.^83,84^ Success of durable repair should be greater than 95%. The most important factor of durable success for mitral repair is the experience of the surgeon.^85,86^

- Type I mitral regurgitation management: If it is due to annular dilation it can be repaired with an annuloplasty ring, with the goal to reduce annular dilation and increase the coaptation zone.^87,88^ Type I mitral regurgitation due to endocarditis may also be repaired depending on the amount of leaflet destruction. A small perforation can be patched, while larger lesions may require resection or plication.^89^
- Type II mitral regurgitation management: Mitral valve repair is the preferred treatment for primary degenerative mitral regurgitation. The feasibility of repair depends on the presence of repairable pathology, which is most likely with excess length and mobility.^90^ Mitral valve repair for degenerative disease consists of reconstruction of the valve, which is usually accompanied by an annuloplasty ring.^91,92^ Prolapsed valves are usually treated with resection of the prolapsing segment(s) and plication of the annulus. For the most common form of posterior leaflet prolapse, different repair techniques can be used alone or in combination. These include resection of the prolapsing segment (s),^93^ plication,^94^ folding-plasty,^95^ sliding leaflet-plasty^96^ or an Alfieri repair,^97^ which is a leaflet edge-to-edge suturing.
- Type III mitral regurgitation management: Patients with rheumatic disease are usually managed with valve replacement. Management of Type IIIb regurgitation involves the use of an isolated annuloplasty, which accomplishes the immediate goal of repair, but without long- term durability, as the ventricular dysfunction and remodeling are not addressed with the annuloplasty.^98,99^ Studies show a more reliable reduction in mitral regurgitation with valve replacement,^100^ with no difference in major outcomes between repair and replacement.^99,101^

### D. Watchful waiting versus early surgical intervention in asymptomatic patients with severe mitral regurgitation

High volume centers of excellence are moving toward a more aggressive surgical approach and they recommend mitral valve repair in asymptomatic patients with severe mitral regurgitation in the absence of left ventricular dysfunction, atrial fibrillation and pulmonary hypertension. That is a Class IIa indication in the 2014 ACC/ AHA guidelines^6^ and a Class IIb in the 2012 European guidelines.^72^ The only required condition for that recommendation is a likelihood of successful repair >95% with low associated mortality <1%.^84–86^

There has been continuous debate in regards to the optimal timing of surgery for asymptomatic patients with severe primary regurgitation who have normal left ventricular systolic function and dimensions, normal pulmonary artery pressure and no episodes of atrial fibrillation. The supporters of the watchful waiting, or “wait and see”, approach argue that surgery should be delayed until Class I or Class IIa indications occur, while the early surgical intervention side supports mitral valve repair before symptoms or any complication of mitral regurgitation happen.

Rosenhek *et al.* reported excellent results with the watchful waiting strategy.^52^ The group examined 132 asymptomatic patients with severe degenerative mitral regurgitation due to flail leaflets or valve prolapse. The patients had close clinical and echocardiographic follow-up, at least every 12 months and in some case every 3-6 months and were referred for surgery when a Class I or a IIa trigger was reached. The overall survival at 8 years was 91 ± 3%, which was not different from the expected survival.^52^

In contrast to the above findings, three groups compared the watchful waiting strategy to the early surgical intervention and showed an advantage of early intervention in terms of long term and operative mortality, as well as repair rates.

Kang *et al.* studied 447 asymptomatic patients with severe degenerative mitral regurgitation and preserved left ventricular function.^102^ 161 patients were referred for early surgery and 286 were managed with the “wait and see” strategy. They showed that the group treated with early surgery had an improved 7- year event free survival (99% ± 1% versus 85% ± 4% for the watchful waiting group, p = 0.001) and less hospitalization for heart failure.^102^

Montant *et al.* showed similar results following prospectively 192 asymptomatic patients with severe degenerative mitral regurgitation.^103^ 67 patients were managed with the conservative approach, while 125 patients underwent early surgical intervention. The 10- year overall survival of the group treated with the conservative strategy was significantly lower (50 ± 7%) compared to the group that received early surgery (86 ± 4%, p < 0.0001). Subgroup analysis of patients with atrial fibrillation and pulmonary hypertension demonstrated similar findings.^103^

Suri *et al.* studied 1021 patients with mitral regurgitation due to flail leaflet that were asymptomatic and had normal LVEF and dimensions.^104^ 575 patients were managed conservatively and 446 were referred to surgery within 3 months of diagnosis. Early mitral valve intervention was associated with significant long -term survival benefit and reduced heart failure risk.^104^

A recent meta- analysis supported an early surgical intervention in asymptomatic severe mitral regurgitation, as it improves survival and increases the likelihood of successful mitral valve repair compared to the watchful waiting strategy.^105^

In summary, current data favors early mitral valve repair in high volume centers able to achieve high success rate, with low procedural mortality. This is more applicable in the isolated posterior mitral valve prolapse (fibroelastic deficiency) and less likely successful in the Barlow’s disease.

## Conclusions

Mitral regurgitation is the most common valvular disorder in the USA and the second most common in Europe. Myxomatous degeneration of the valve is responsible for two thirds of primary mitral regurgitation, which is an intrinsic valve problem in contrast to the secondary regurgitation, which is a disease of the ventricle. Mitral regurgitation is a pure volume overload to the left ventricle, to which the ventricle responds with progressive dilation and eventually with decreased ejection fraction and symptoms of heart failure. Quantitative echocardiography is the main way to evaluate the severity of mitral regurgitation. Cardiac MRI and left/right heart catheterization have an adjunctive role when there is discrepancy between clinical findings and echocardiographic data. Clinical (age, symptoms, poor exercise capacity, atrial fibrillation) and echocardiographic (left ventricular dysfunction, EROA, high SPAP and left atrial volume and abnormal LV strain) parameters predict worse outcomes in mitral regurgitation. Patients with severe primary regurgitation have a high morbidity and mortality rate and in 10 year follow-up 90% will have died or undergone surgery due to development of symptoms. Surgical intervention, preferably valve repair, is indicated in patients with severe primary mitral regurgitation and symptoms or evidence of left ventricular dysfunction, defined as ejection fraction <60% and end-systolic dimension >40 mm. Patients with severe asymptomatic mitral regurgitation can be managed with meticulous follow-up for symptoms and left ventricular dysfunction, but studies appear to favor early valve repair in centers of excellence able to achieve high repair rates (>95%) and low mortality (<1%) especially for patients with localized prolapse. Transcatheter mitral valve repair techniques have emerged in recent years and appear to be safe, especially in elderly people with extensive comorbidities who are frequently denied surgery.^106^ The future of percutaneous options may change the threshold for intervention and requires careful assessment.

## Conflict of interests

The authors have no competing interests to declare.

**Authors contributions**

EA: contributed to the design of the review, drafted the original manuscript and gave final approval.

ADM: contributed to the concept, created the movies, critically revised the content of the manuscript and gave final approval.

AP: contributed to concept and design of the review, critically revised the content of the manuscript and gave final approval.

The authors read and approved the final manuscript.
